# NO Deficiency Compromises Inter- and Intrahemispheric Blood Flow Adaptation to Unilateral Carotid Artery Occlusion

**DOI:** 10.3390/ijms25020697

**Published:** 2024-01-05

**Authors:** László Hricisák, Éva Pál, Dorina Nagy, Max Delank, Andreas Polycarpou, Ágnes Fülöp, Péter Sándor, Péter Sótonyi, Zoltán Ungvári, Zoltán Benyó

**Affiliations:** 1 Institute of Translational Medicine, Semmelweis University, 1094 Budapest, Hungary; hricisak.laszlo@med.semmelweis-univ.hu (L.H.); pal.eva@med.semmelweis-univ.hu (É.P.); dorinanagy1011@gmail.com (D.N.); maxdelank@gmail.com (M.D.); polyc003@UMN.edu (A.P.); fulop.agnes@med.semmelweis-univ.hu (Á.F.); drsanpet@gmail.com (P.S.); 2 HUN-REN-SU Cerebrovascular and Neurocognitive Diseases Research Group, 1094 Budapest, Hungary; 3 Mayo Clinic, College of Medicine and Science, Rochester, MN 55905, USA; 4 Division of Cardiothoracic Surgery, University of Minnesota, Minneapolis, MN 55455, USA; 5 Department of Vascular and Endovascular Surgery, Semmelweis University, 1122 Budapest, Hungary; sotonyi.peter1@med.semmelweis-univ.hu; 6 Vascular Cognitive Impairment, Neurodegeneration and Healthy Brain Aging Program, Department of Neurosurgery, University of Oklahoma Health Sciences Center, Oklahoma City, OK 73104, USA; zoltan-ungvari@ouhsc.edu; 7 Department of Health Promotion Sciences, College of Public Health, University of Oklahoma Health Sciences Center, Oklahoma City, OK 73104, USA; 8 International Training Program in Geroscience, Doctoral College/Department of Public Health, Semmelweis University, 1089 Budapest, Hungary; 9 The Peggy and Charles Stephenson Cancer Center, University of Oklahoma Health Sciences Center, Oklahoma City, OK 73104, USA

**Keywords:** nitric oxide synthase, cerebral autoregulation, carotid artery stenosis

## Abstract

Carotid artery stenosis (CAS) affects approximately 5–7.5% of older adults and is recognized as a significant risk factor for vascular cognitive impairment (VCI). The impact of CAS on cerebral blood flow (CBF) within the ipsilateral hemisphere relies on the adaptive capabilities of the cerebral microcirculation. In this study, we aimed to test the hypothesis that the impaired availability of nitric oxide (NO) compromises CBF homeostasis after unilateral carotid artery occlusion (CAO). To investigate this, three mouse models exhibiting compromised production of NO were tested: NOS1 knockout, NOS1/3 double knockout, and mice treated with the NO synthesis inhibitor L-NAME. Regional CBF changes following CAO were evaluated using laser-speckle contrast imaging (LSCI). Our findings demonstrated that NOS1 knockout, NOS1/3 double knockout, and L-NAME-treated mice exhibited impaired CBF adaptation to CAO. Furthermore, genetic deficiency of one or two NO synthase isoforms increased the tortuosity of pial collaterals connecting the frontoparietal and temporal regions. In conclusion, our study highlights the significant contribution of NO production to the functional adaptation of cerebrocortical microcirculation to unilateral CAO. We propose that impaired bioavailability of NO contributes to the impaired CBF homeostasis by altering inter- and intrahemispheric blood flow redistribution after unilateral disruption of carotid artery flow.

## 1. Introduction

Internal carotid artery stenosis (CAS) is a prevalent steno-occlusive arterial disease, affecting approximately 5% to 7.5% of older adults [1], and represents a significant risk factor for the development of vascular cognitive impairment (VCI) [2,3,4,5,6,7,8]. CAS is characterized by the atherosclerotic narrowing of the carotid artery, resulting in reduced blood supply to the ipsilateral hemisphere of the brain. Atherosclerotic CAS is a leading risk factor for ischemic stroke, which is a major cause of mortality and long-term disability in developed countries [9]. The mechanisms by which CAS promotes cerebral ischemic events are thought to involve hemodynamic changes in addition to thromboembolism [10,11,12,13,14]. The magnitude of decline in cerebral blood flow (CBF) within the ipsilateral hemisphere, and thereby the clinical consequences of CAS, rely on the adaptive capabilities of the cerebral microcirculation [15]. Compensatory mechanisms involving inter- and intrahemispheric blood flow redistribution are crucial to maintain the blood supply in the affected ipsilateral region and to prevent severe brain ischemia.

To prevent stroke, eligible patients with significant CAS are treated with carotid endarterectomy [16,17]. Dysregulation of CBF in these patients contributes to perioperative complications during the procedure [18,19,20]. During carotid endarterectomy, the temporary cessation of carotid artery flow is necessary for plaque removal or artery repair, posing a risk of brain ischemia, particularly in the affected hemisphere. The duration of this critical period ranges from 20 to 30 min, depending on the procedure’s complexity. Impaired compensatory CBF adaptation to flow cessation in the carotid artery can contribute to perioperative complications and increase the risk of ischemic events.

Understanding how the cerebrocortical microcirculation adapts to compromised blood flow is critical for improving surgical procedures and patient outcomes. Factors that influence adequate hemodynamic compensation and the clinical consequences of CAS as well as the complications of carotid endarterectomy involve the robustness of the collateral circulation [5,12,21,22,23,24,25,26,27,28], compensatory inter- and intrahemispheric blood flow redistribution, and the effectiveness of cerebral microcirculatory blood flow autoregulation [10,11,12,13,14,21,22,29,30,31,32]. Investigating these mechanisms is vital for optimizing patient care and reducing the risk of adverse events during carotid artery interventions.

The availability of nitric oxide (NO), a potent vasodilator produced by endothelial cells and neurons, plays a critical role in regulating CBF and maintaining cerebrovascular homeostasis [2,33,34,35,36,37,38,39,40,41,42]. Impaired bioavailability of NO, associated with advanced aging and comorbidities such as hypertension, diabetes mellitus, vitamin D deficiency, and cardiovascular disease [43,44,45], is likely to contribute to compromised CBF homeostasis in older patients at risk for VCI. Understanding the combined impact of CAS and these comorbidities on the compensatory mechanisms and adaptive responses of the cerebral microcirculation is crucial in elucidating the clinical consequences of CAS. However, the specific role of impaired NO mediation in compensatory CBF adaptation during carotid artery blood flow cessation remains incompletely understood.

In light of these considerations, this study aimed to investigate the role of NO in cerebrovascular adaptation in a model of unilateral carotid artery flow cessation. To achieve this, three preclinical mouse models of impaired NO synthesis were utilized: NOS1 knockout (NOS1 KO) mice, NOS1 and NOS3 double knockout (NOS1/3 DKO) mice, and mice treated with L-NAME, an inhibitor of NO synthesis. The effects of unilateral carotid artery occlusion (CAO) on ipsi- and contralateral regional hemispheric blood flow were assessed using laser-speckle contrast imaging (LSCI). Morphological changes in pial anastomoses, which play a role in collateral circulation, were also examined. Based on previous evidence, we hypothesized that impaired availability of vasodilator NO compromises CBF homeostasis by altering inter- and intrahemispheric blood flow redistribution after the unilateral disruption of carotid artery flow.

## 2. Results

### 2.1. Body Measures

Table 1 presents the body parameters of wild type and genetically modified mice. Notably, NOS1 KO and NOS1/3 DKO mice exhibited significantly smaller body, heart, and brain weights compared to wild type animals. Interestingly, a proportional decrease in body weight resulted in an unchanged heart weight/body weight ratio. Similarly, as the tibial lengths were comparable, the ratios of body weight/tibial length, heart weight/tibial length also decreased in NOS1 KO and NOS1/3 DKO mice. However, the brain weight exhibited a lesser extent of decrease compared to body weight in these mice, leading to an increased brain weight/body weight ratio when compared to wild type animals. NOS1 KO animals express a prominent phenotype of gastric and bowel enlargement, likely attributable to both pyloric hypertrophy and dysmotility, as NOS1 is reported to regulate gastrointestinal motility, as well [46]. Thus, the aforementioned differences in body parameters likely can be attributed to NOS1 deficiency-induced gastrointestinal dysfunction and subsequent underdevelopment.

### 2.2. Morphological Parameters of Leptomeningeal Collaterals

**Figure 1:**
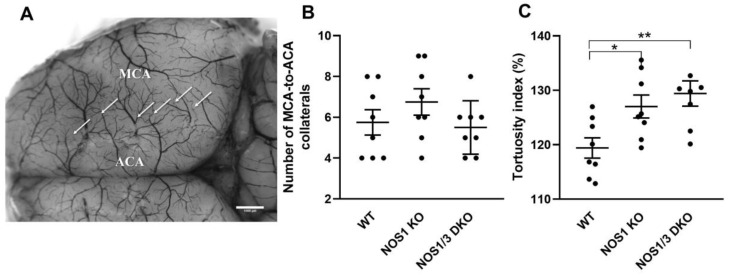
Morphology of leptomeningeal collaterals in wild-type (WT), NOS1 KO, and NOS1/3 DKO mice. Panel (**A**) shows a representative image of the cerebrocortical vessels in a control mouse brain. The arrows indicate the leptomeningeal collaterals between the territories of the middle cerebral artery (MCA) and anterior cerebral artery (ACA). (**B**) Number of MCA-to-ACA collaterals. No significant difference in the number of collaterals is observed between the groups (*p* = 0.2963; one-way Anova). (**C**) Tortuosity index. The tortuosity index is significantly increased in NOS1 KO (* *p* = 0.0474) and NOS1/3 DKO animals (** *p* = 0.0082) compared to controls (one-way Anova with Tukey’s post hoc test). Scatter dot plots of hemispheres with mean ± SEM are shown.

Given the significant role of leptomeningeal collateral vessels in the adaptive response of the cerebral circulation to changes in blood supply from major cerebral vessels, such as the internal carotid and basilar arteries, we sought to investigate whether the deletion of NOS1 and NOS3 genes results in altered morphological properties of pial collaterals. Hence, we examined both the number and tortuosity of these collaterals (Figure 1).

Figure 1B illustrates that there was no significant difference in the number of collaterals between the experimental groups. However, the tortuosity index of NOS1 KO and NOS1/3 DKO animals was found to be significantly increased compared to the control group (Figure 1C). This finding suggests a potential impairment in adaptation due to the increased tortuosity of vessels connecting the two cortical regions.

### 2.3. Acid-Base, Blood gas, Hematocrit, and Electrolyte Parameters

The acid-base, blood gas, hematocrit, and electrolyte parameters were assessed at the end of the experiments using femoral arterial samples, and the results are presented in Table 2. Importantly, all of these physiological parameters were similar in the different experimental groups and remained within the normal range. The presence of a mild metabolic acidosis, observed in all experimental groups, is likely attributable to the effects of anesthesia [47].

### 2.4. Blood Pressure

**Figure 2:**
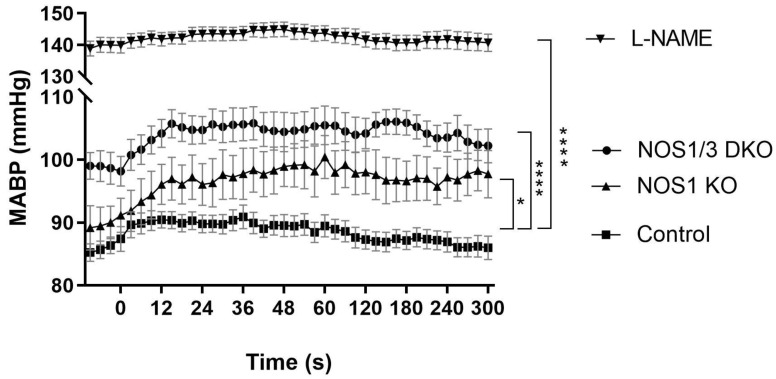
Mean arterial blood pressure (MABP) in control wild type, NOS1 KO, NOS1/3 DKO, and L-NAME-treated wild-type mice throughout the in vivo experiments. The zero point on the *x*-axis represents the time of carotid artery occlusion. NO deficiency resulted in elevated blood pressure in the NOS1 KO, NOS1/3 DKO, and L-NAME-treated mice as compared to the control group (* *p* = 0.0218, **** *p* < 0.0001, two-way Anova). The values are presented as mean ± SEM. The number of animals (*n*) in each group is 10, 6, 11, and 15 for the Control, NOS1 KO, NOS1/3 DKO, and L-NAME groups, respectively.

Given the pivotal role of NO in the regulation of vascular tone [48,49], systemic blood pressure was assessed in all experimental groups throughout the experiments. In NOS1 KO mice, a moderate elevation of blood pressure was observed compared to the control group (Figure 2). In contrast, a marked hypertension was evident in NOS1/NOS3 DKO mice (Figure 2), consistent with the idea that eNOS-derived NO regulates peripheral vascular resistance [48,50]. Pharmacological inhibition of NO synthesis led to an even more robust increase in blood pressure, with average values reaching approximately 140 mmHg in this group. Interestingly, while the control, NOS1 KO, and NOS1/3 DKO animals displayed a slight increase in blood pressure upon carotid artery occlusion at 0 s, this response was completely absent in the already hypertensive L-NAME group.

### 2.5. Changes in Regional CoBF after Unilateral CAO

**Figure 3:**
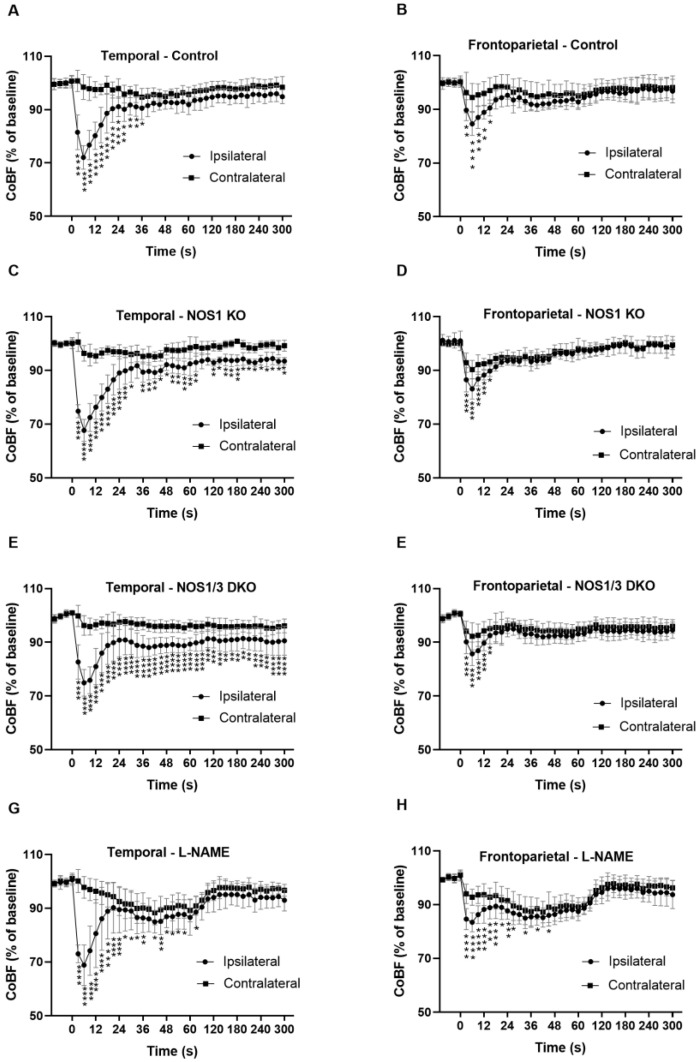
Cerebrocortical blood flow (CoBF) in the ipsilateral and contralateral hemispheres after unilateral carotid artery occlusion (CAO). Panels A and B: Blood flow changes in temporal (**A**) and frontoparietal (**B**) regions in control mice (*n* = 10) after unilateral CAO. In both ipsilateral regions, the blood flow normalizes after a transient reduction post-CAO. Panels C and D: Blood flow changes in temporal (**C**) and frontoparietal (**D**) regions in NOS1 KO animals (*n* = 6) after unilateral CAO. Note the less complete recovery in the ipsilateral temporal region compared to the frontoparietal region. Panels E and F: Blood flow changes in temporal (**E**) and frontoparietal (**F**) regions in NOS1/3 DKO animals (*n* = 11) after unilateral CAO. Note the sustained hypoperfusion in the temporal region but not in the frontoparietal region. Panels G and H: Blood flow changes in temporal (**G**) and frontoparietal (**H**) regions of L-NAME-treated animals (*n* = 15) after unilateral CAO. Note that acute severe hypoperfusion is both regions of the ipsilateral hemisphere and mild hypoperfusion of the contralateral hemisphere in the first 90 s. after CAO and the recovery of CoBF in all regions thereafter. Values are presented as mean ± SD percentage of the baseline. Circles indicate the ipsilateral, while squares indicate the contralateral hemisphere. Statistical significance is denoted as * *p* < 0.05, ** *p* < 0.01, *** *p* < 0.001, **** *p* < 0.0001 vs. contralateral (two-way Anova with Bonferroni’s post hoc test).

The changes in cerebrocortical blood flow (CoBF) following unilateral common carotid artery occlusion were compared between the ipsilateral and contralateral hemispheres in different experimental groups, including the control, genetically modified (NOS1 KO, NOS1/3 DKO), and pharmacologically treated (with L-NAME) mice (Figure 3). Figure 3A,B show the CoBF changes in wild type control mice. The most pronounced acute CoBF reduction (−30%) after CAO appeared in the ipsilateral temporal region (Figure 3A). This is reasonable because the temporal cortex receives its blood supply predominantly from the middle cerebral artery (MCA), which originates from the circle of Willis close to its junction with the internal carotid artery. Thereafter, the CoBF of this region recovered to 90% of its original level within 30 s, indicating the effective acute adaptation of the cerebral circulation to CAO, and remained slightly below the baseline level in the remaining part of the experiment. In contrast, the blood flow of contralateral temporal cortex remained practically at the baseline level (Figure 3A). The acute CoBF reduction was less pronounced (−15%) in the ipsilateral frontoparietal region and also returned to 90% of its original level within 30 s, whereas the contralateral frontoparietal cortex did not show any marked hypoperfusion (Figure 3B). The transient difference in the blood flow of the ipsilateral vs. contralateral frontoparietal regions is an important finding because both of these territories are supplied by the common anterior cerebral artery (ACA). Therefore, the more pronounced CoBF reduction in the ipsilateral side indicates that the blood is drained through pial collaterals to the more ischemic temporal cortex in order to prevent severe hypoxia.

In NOS1 KO animals, in the ipsilateral temporal region, the acute CoBF drop and its fast recovery within the first 30 s were similar to controls, but the blood flow remained significantly lower than that of the contralateral temporal cortex throughout the whole experiment (Figure 3C), indicating the involvement of NOS1 in the adaptation of the cerebral circulation to CAO. The mild hypoperfusion in the temporal region of NOS1 KO mice may be attributed to less effective pial collateral circulation due to the presence of more tortuous anastomoses as shown in Figure 1C. Nevertheless, the blood flow in the temporal region was able to recover to almost 90% of the baseline level, indicating partially reserved ability for adaptation. The temporal pattern of the CoBF changes in the frontoparietal regions of NOS1 KO mice (Figure 3D) was very similar to that of the control animals (Figure 3B), and significant differences between the two hemispheres appeared also only in the acute phase (up to 15 s). However, in the temporal region of NOS1 KO mice (Figure 3C), significant differences between the hemispheres were evident at nearly all time points.

To further examine the role of NO in the adaptation mechanism, mice with genetic deficiency of both NOS1 and NOS3 were tested (Figure 3E,F). In this mouse model, significant differences between the two hemispheres were consistently present in the temporal region throughout the entire measurement (Figure 3E). Although a marked recovery was also present during the first 30 s, the blood flow in the ipsilateral temporal region remained below 90% of the baseline until the end of the experiment, indicating a worsened adaptational capacity in the double knockout animals. Interestingly, the CoBF in the contralateral temporal cortex also remained below the baseline after CAO throughout the entire experiment (Figure 3E). In the frontoparietal regions, the CoBF changes in the acute phase were similar to those observed in control and NOS1 KO animals with interhemispheric differences during the first 15 s (Figure 3F). However, in contrast to other experimental groups, the blood flow of both the ipsilateral and the contralateral frontoparietal regions remained below the baseline during the subacute phase until the end of the measurements (Figure 3F).

Figure 3G,H depict the CoBF changes in the temporal and frontoparietal regions of L-NAME-treated animals. Despite the complete inhibition of NO synthesis in this group, the immediate maximal blood flow reduction was not enhanced by L-NAME pretreatment. However, the recovery of blood flow was delayed in both ipsilateral regions, resulting in a more severe hypoperfusion. Interestingly, a significant reduction in CoBF was observed in both regions of the contralateral hemisphere of L-NAME-treated animals during the acute phase after CAO (Figure 3G,H). In the subacute phase, approximately 1 min after the occlusion, the blood flow started to increase and reached or even exceeded 90% of the baseline level in all investigated regions of the ipsilateral and the contralateral hemispheres (Figure 3G,H).

**Figure 4:**
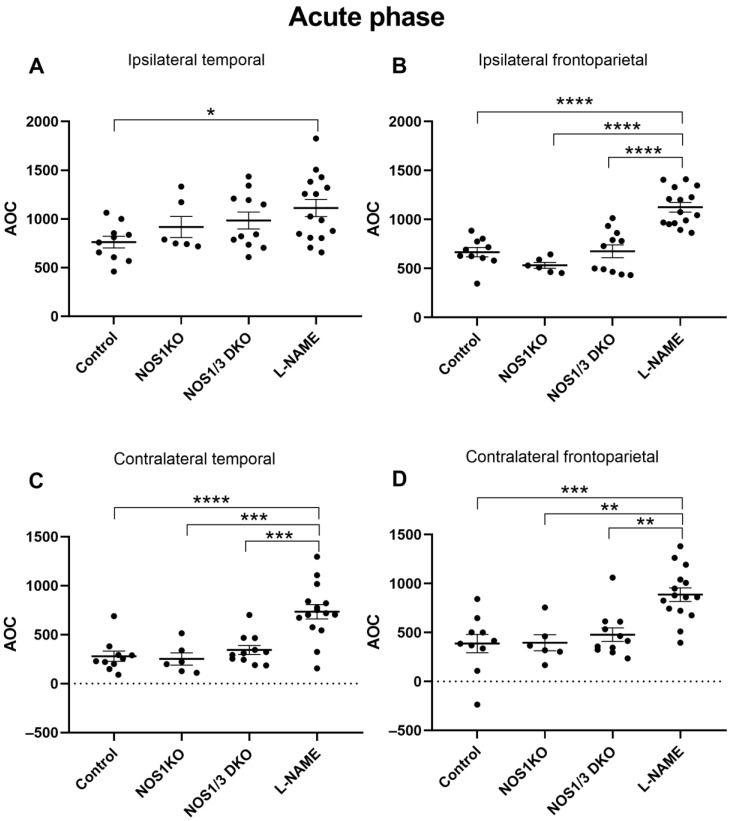
Comparison of hypoperfusion levels determined by AOC (area over the curve) values of blood flow in control wild type, NOS1 KO, NOS1/3 DKO, and L-NAME-treated wild-type mice in the acute phase (6–90 s) after unilateral carotid artery occlusion. L-NAME-treated animals exhibit a significant difference compared to controls in the ipsilateral temporal region (Panel (**A**), * *p* = 0.0241), as well as compared to all other groups in the ipsilateral frontoparietal region (Panel (**B**), **** *p* < 0.0001 vs. all groups). Furthermore, L-NAME-treated animals display a significant difference both in the contralateral temporal (Panel (**C**), *** *p* < 0.001, **** *p* < 0.0001) and frontoparietal regions (Panel (**D**), ** *p* < 0.01, *** *p* < 0.001) compared to all other groups. Scatter dot plots with mean ± SEM are shown. Statistical analysis was performed with one-way Anova and Tukey’s post hoc test.

**Figure 5:**
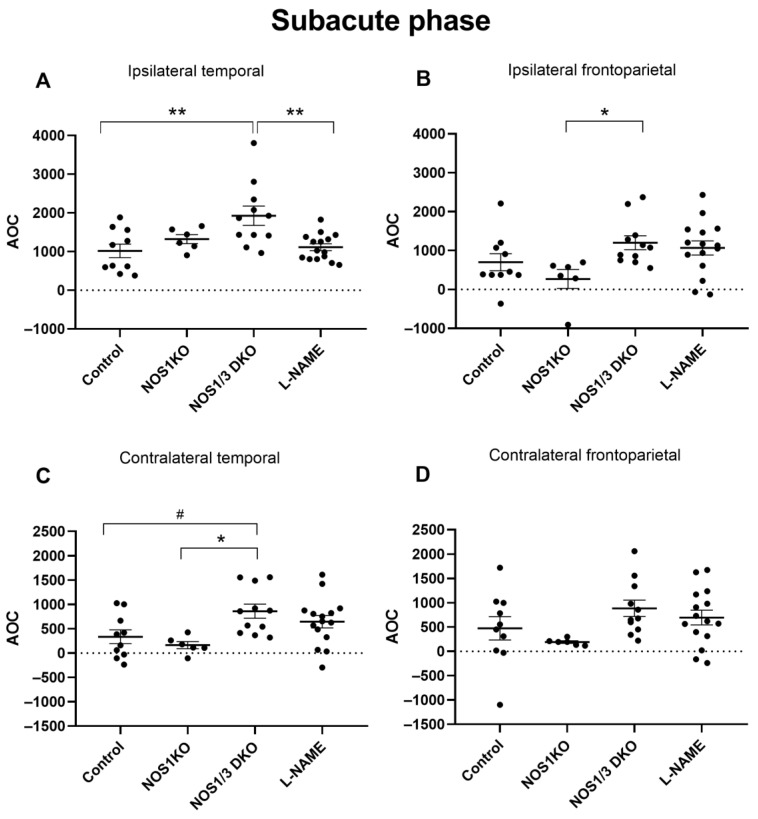
Comparison of hypoperfusion levels determined by AOC (area over the curve) values of blood flow in control wild type, NOS1 KO, NOS1/3 DKO, and L-NAME-treated wild-type mice in the subacute phase (90–300 s) after unilateral carotid artery occlusion. NOS1/3 DKO animals show worsened recovery in the ipsilateral temporal (Panel (**A**), ** *p* < 0.01) and frontoparietal (Panel (**B**), * *p* = 0.0400) regions. The blood flow recovery of NOS1/3 DKO mice is also compromised in the temporal (Panel (**C**), * *p* = 0.0208 and ^#^ *p* = 0.0514) region, but not in the frontoparietal region (Panel (**D**)), of the contralateral hemisphere. Scatter dot plots with mean ± SEM are shown. Statistical analysis was performed with one-way Anova and Tukey’s post hoc test).

After assessing the temporal patterns of CoBF changes and the differences between the ipsilateral and contralateral regions following unilateral CAO, our next objective was to quantify the overall CoBF deficits during the acute phase (up to 90 s, Figure 4) and subacute phase (90–300 s, Figure 5) of adaptation. Figure 4 presents the area above the curve (AOC) values in the acute phase of the adaptation, which reflect the magnitude of CoBF changes, in the frontoparietal and temporal regions of the ipsilateral and contralateral hemispheres in the control, NOS1 KO, NOS1/3 DKO, and L-NAME-treated animals. The pharmacological inhibition of NO synthesis using L-NAME resulted in the most pronounced CoBF reductions in the ipsilateral hemispheres during the acute phase. Significant differences were observed in the ipsilateral temporal region between control and L-NAME-treated animals (Figure 4A), as well as between L-NAME-treated animals and all other groups in the ipsilateral frontoparietal region (Figure 4B). Furthermore, in the contralateral hemisphere, the L-NAME-treated animals showed a significantly increased CoBF reduction as compared to all other experimental groups both in the temporal (Figure 4C) and in the frontoparietal (Figure 4D) regions.

In the subacute phase (90–300 s after CAO, Figure 5A–D), NOS1/3 DKO animals exhibited an enhanced blood flow deficit in the ipsilateral temporal region compared to controls (Figure 5A), indicating a compromised adaptational capacity after unilateral CAO. Interestingly, a significant difference was also observed between the NOS1/3 DKO and NOS1 KO groups in the ipsilateral frontoparietal (Figure 5B) and contralateral temporal region (Figure 5C), where the difference between control and NOS1/3 DKO animals also almost reached the level of statistical significance (Figure 5C). Taken together, immediately after the occlusion (acute phase), the complete pharmacological NOS blockade by L-NAME induced the most significant adaptation deficit and cerebrocortical hypoperfusion, whereas during the subacute phase (90–300 s after CAO), NOS1/3 DKO animals showed compromised adaptation and prolonged CoBF reduction, further highlighting the interaction between the two NOS isoforms in the cerebrovascular system.

## 3. Discussion

In the present study, we focused on assessing the role of NO and NO synthase isoforms, which are known to play key roles in regulating cerebral vascular tone and blood flow [40,41,42,51,52,53,54], in the adaptation of cerebrocortical microcirculation to unilateral CAO. This experimental model closely mimics the hemodynamic conditions observed in patients with CAS undergoing carotid endarterectomy. By investigating the effects of NO and NO synthase isoforms, we aimed to shed light on the underlying mechanisms involved in cerebrovascular adaptation during reduced blood supply to the brain. We utilized genetically modified mouse models and pharmacological interventions to assess the contribution of NO synthase isoforms to cerebrovascular adaptation following CAO.

Our findings suggest that NOS1 plays a role in the adaptational process, as mice lacking neuronal nitric oxide synthase (NOS1 KO) and double knockout mice of NOS1 and endothelial nitric oxide synthase (NOS1/3 DKO) exhibited impaired cerebrocortical blood flow adaptation to CAO, particularly in the subacute phase. The more pronounced impairment observed in NOS1/3 DKO mice indicates a synergistic role of NO derived from NOS1 and NOS3 in maintaining cerebrovascular homeostasis. Interestingly, previous studies reported that genetic depletion of NOS3 alone does not significantly impair CBF adaptation during CAO, likely due to up-regulation of compensatory pathways such as vasodilator prostanoid synthesis [55]. In the present study, pharmacological inhibition of NO synthesis resulted in severe alterations in CBF. L-NAME-treated animals exhibited a marked and prolonged hypoperfusion in both the ipsilateral and contralateral hemispheres in the acute phase, confirming a crucial role of NO in compensatory mechanisms following CAO. An interesting finding of the present study is that whereas pharmacological inhibition of NO synthesis had more severe consequences in the acute phase of adaptation, genetic deletion of NOS1/3 mostly compromised the CBF recovery during the subacute phase. We propose that this difference may be related to the compensatory vasoregulatory mechanisms that are upregulated during chronic NO deficiency in the NOS1/3 DKO animals, and probably explain the milder hypertension in NOS KO compared to L-NAME-treated mice. These compensatory mechanisms may support the CBF recovery during the acute phase of adaptation to CAO resulting in a less severe phenotype of NOS1/3 DKO as compared to L-NAME-treated mice. However, in the subacute phase the unfavorable morphological change (increased tortuosity) of the leptomeningeal collaterals and lack of NO-mediated vasodilation together may be responsible for the prolonged hypoperfusion of the brain in NOS1/3 DKO mice. Further studies are warranted to investigate whether similar impairments in cerebrocortical blood flow adaptation mechanisms to CAO are present in animal models of various cardiovascular risk factors, including diabetes, which are known to be characterized by oxidative stress, impaired NO bioavailability, and endothelial dysfunction. This would provide valuable insights into the specific contributions of impaired NO mediation to the observed cerebrovascular dysfunction and shed light on potential therapeutic targets for preserving cerebrovascular homeostasis and preventing perioperative complications in CAS patients with cardiovascular risk factors.

During carotid artery occlusion, the main collaterals that help maintain interhemispheric blood flow balance include the circle of Willis and the secondary cerebral collateral system, including leptomeningeal, pial [55], and extra-intracranial arterial anastomoses [56]. NO is an important regulator of angiogenesis [57,58]. The morphological alterations observed in pial collaterals in the NOS-deficient animals provide initial evidence for the involvement of NO produced by both NOS1 and NOS3 in regulating both the morphology and functionality of the cerebrocortical microcirculation. Further studies are needed to evaluate the differential effects of chronic NO deficiency on each collateral system.

The present study provides valuable insights into the role of NO and NO synthase isoforms in the adaptation of cerebrocortical microcirculation to unilateral CAO and their potential contribution to the pathophysiology of CAS and perioperative complications during carotid endarterectomy. However, several limitations should be considered. Firstly, this study focused on the specific role of NOS1 and NOS3 in cerebrovascular adaptation, and further investigations are needed to elucidate the underlying cellular mechanisms. Interestingly, morphological and functional studies indicate that the neuronal NOS1 isoform is present in the cerebrovascular endothelium and contributes to the regulation of the vascular tone [40,59]. Exploring the role of NO in the neurovascular unit, potential interactions with other vasoregulatory mechanisms, and the involvement of specific cell types within the cerebrovascular system would provide a more comprehensive understanding of NO-mediated compensatory responses. Additionally, the study primarily utilized genetically modified mouse models and pharmacological inhibition of NO synthesis. While these models provide valuable insights into the specific contributions of NO and NOS isoforms, it is essential to extend the research to animal models of aging [33,36,60,61] and models that mimic various cardiovascular risk factors, such as diabetes and metabolic disease, which are known to be characterized by endothelial dysfunction and impaired NO bioavailability [43]. This would allow for a more comprehensive assessment of the role of NO in cerebrovascular adaptation under conditions relevant to older CAS patients with comorbidities.

### Implications of the Results in a Clinical Setting

In terms of clinical implications, the findings of this study highlight the potential significance of impaired NO mediation in CAS patients with cardiovascular risk factors. Clinical studies aimed at understanding the specific contributions of NO deficiency and endothelial dysfunction to cerebrovascular impairments, altered CBF adaptation to carotid artery occlusion, and perioperative complications are warranted. Future studies should also investigate the synergistic actions of aging and comorbidities [62,63,64].

Our present findings have an important relevance related to the surgical interventions in the carotid artery. Whereas atherosclerotic stenosis or occlusion of the carotid artery usually develops slowly (within months or years), allowing remodeling of the collateral circulation, during carotid surgery, the acute cessation of the carotid arterial blood flow is often necessary, and the maintenance of sufficient perfusion of the brain depends on the preexisting collateral vessels as well as on the acute compensatory blood flow redistribution mechanisms, which were in the focus of the present study. The former can be considered as the anatomical whereas the latter can be considered as the functional modality of adaptation in order to preserve the oxygen and nutrient supply of the brain. Before carotid endarterectomy, which involves the temporal occlusion of the artery, the morphological parameters of the collateral network are determined by computed tomography angiography (CTA) or transcranial Doppler ultrasound [65,66]. As indicated in our previous study, completeness and effectiveness of the circle of Willis (CoW) are of key importance regarding the compensatory CBF adaptation, and missing branches of the CoW impairs the brain functions after carotid artery clamping [65]. However, even the most precise mapping of the collateral vascular connections cannot unambiguously predict the consequences of carotid artery occlusion on brain oxygenation, which indicates the importance of the functional adaptation mechanisms of the cerebral vessels. Therefore, during carotid surgery, the adaptation capacity is also examined by evaluating the oxygen supply of the brain after clamping of the carotid artery, either directly (with near-infrared spectroscopy, NIRS) or indirectly with stump pressure or systemic arterial blood pressure measurement (appearance of Cushing reflex). In case of an incomplete CoW or improper adaptation capacity, a Le Maitre shunt is indicated to support the collateral circulation during endarterectomy (selective shunting) [67]; however, this intervention can increase the surgical burden and may itself lead to cerebral embolism. Our present study was focusing on the functional adaptation mechanism of the cerebral circulation to acute carotid artery occlusion and identified NO as an important mediator in this process. Preoperative evaluation and eventual new therapies for supporting this functional modality of cerebrovascular adaptation may have a major impact in prevention of cerebral hypoxia and its neurological consequences during carotid artery surgery. Addressing these aspects will contribute to a more comprehensive understanding of cerebrovascular pathophysiology and facilitate the development of tailored strategies for CAS management and perioperative care.

## 4. Materials and Methods

### 4.1. Experimental Animals

Male mice with a C57Bl6/N genetic background, aged between 12 and 17 weeks, served as control animals in this study. To investigate the role of nitric oxide synthases (NOS) after unilateral common carotid artery occlusion, two genetically modified mouse models were employed: NOS1 knockout (NOS1 KO, neuronal NOS knockout) and NOS1/3 double knockout (NOS1/3 DKO, neuronal and endothelial NOS double knockout). Additionally, a pharmacological approach was employed, involving the use of L-NAME (N-nitro-L-arginine methyl ester) treatment in control animals to inhibit NO synthesis by all NOS isoforms. The animals were housed in a specific pathogen-free animal facility with a 12/12 h dark/light cycle, and they had ad libitum access to food and water. All experimental procedures were conducted in accordance with the guidelines of the Hungarian Law of Animal Protection (XXVIII/1998) and adhered to the ARRIVE (Animal Research: Reporting In Vivo Experiments) guidelines. Ethical approval for the study was obtained from the National Scientific Ethical Committee on Animal Experimentation (PEI/001/2706-13/2014, approval date: 17 December 2014; PE/EA/487-6/2021, approval date: 9 November 2021).

### 4.2. Surgical Procedures, Carotid Artery Occlusion

The experimental procedures began with the measurement of the animals’ weight. Subsequently, under 2% isoflurane anesthesia (Aerrane, Baxter Hungary Kft., Budapest, Hungary), the animals were positioned on a heating pad beneath a stereotaxic microscope (Wild M3Z, Heerbrugg, Switzerland). To ensure stable body temperature throughout the experiment, a rectal probe connected to a controlled heating pad was employed. The depth of anesthesia was regularly assessed by monitoring plantar and corneal reflexes. Whenever any signs of pain or arousal were observed, the anesthesia level was deepened accordingly. Following the induction of proper anesthesia, a small incision was made on the left hindlimb, and a PE50 cannula (Intramedic Polyethylene Tubing, Becton Dickinson, Franklin Lakes, NJ, USA) was inserted into the femoral artery. This allowed for the measurement of systemic arterial blood pressure during the experiment and facilitated blood sample collection at the end of the procedure (further details will be provided later). Subsequently, the anesthesia was switched to intraperitoneal administration of ketamine (100 μg/g body weight, Calypsol; Gedeon Richter, Budapest, Hungary) and xylazine (10 μg/g body weight, CP-Xylazine; CP-Pharma, Burgdorf, Germany). This anesthesia regimen was maintained for the remainder of the experiment. To facilitate spontaneous respiration, a tracheal (PE10) cannulation (Intramedic Polyethylene Tubing, Becton Dickinson, Franklin Lakes, NJ, USA) was performed carefully, ensuring no harm was inflicted on the vagus nerve. Proceeding to the left side of the trachea, the carotid sheath was dissected with precision, avoiding contact or damage to the vagus nerve. Once the common carotid artery was exposed, a loop was gently positioned around it in order to be utilized later for occlusion during the experimental procedures.

### 4.3. Measurement of CAO-Induced Changes in CoBF

After the completion of the aforementioned surgical procedures, the animals were positioned in a stereotaxic holder and placed on a heating pad under the laser speckle instrument. Care was taken to secure the head of the animals to prevent any minute movements that could introduce artifacts in the recordings. The femoral artery cannula was connected to a pressure sensor to enable measurement of systemic arterial blood pressure. For assessment of changes in CoBF, the laser speckle contrast imaging (LSCI) method was employed, leveraging its high temporal and spatial resolution. The LSCI instrument used (PeriCam PSI; Perimed, Järfälla, Stockholm, Sweden) was precisely positioned 10 cm above the previously exposed skull, which was accomplished by retracting the skin following a small midline incision. As the initial step of the experimental protocol, atipamezole (1 μg/g ip.; Sigma-Aldrich, St. Louis, MO, USA) was administered as an antidote to counteract the blood pressure-lowering effects of xylazine [55,68]. Following the attainment of stabilized blood pressure, N-nitro-L-arginine methyl ester (L-NAME 10 mg/kg i.p.; Bachem AG, Bubendorf, Switzerland) was administered intraperitoneally to a group of C57Bl6/N wild-type animals, and a 30-min waiting period ensued. Subsequently, baseline measurements of CoBF and blood pressure were obtained, a process taking approximately 10 min. Following these baseline measurements, the unilateral common carotid artery occlusion (CAO) procedure was performed by tightening the previously loosened knot around the vessel. Five minutes subsequent to the occlusion, an arterial blood sample was collected through the cannula into a heparin-coated capillary for subsequent blood gas analysis. The Radiometer ABL80Flex instrument (Radiometer Medical ApS, Brønshøj, Denmark) was employed to assess the blood gas and acid-base parameters. Only experiments with blood gas parameters falling within the physiological range (pO_2_ > 90 mmHg, pCO_2_ between 25 and 55 mmHg) were included in the analysis. To minimize potential impact on blood pressure, blood sampling during the experiment was avoided as it would have necessitated a significant amount of blood, which could have influenced the animals’ hemodynamic status.

The original CoBF data, initially measured in arbitrary units (AU), were subsequently converted into the percentage of the average baseline value recorded over a one-minute period. These percentage values were then averaged at 15-s intervals, including time points −45 s, −30 s, −15 s, and 0 s, the later representing the time of occlusion after the baseline phase. During the first minute following the occlusion, the percentage values were averaged at 3-s intervals. Throughout the remainder of the experiment, the percentage values were averaged every 15 s. The recovery period following the occlusion was divided into two phases: an acute phase spanning from 6 s to 90 s, and a subacute phase spanning from 90 s to 5 min. The analysis of CoBF changes focused on two distinct regions: the frontoparietal and temporal regions, supplied by the anterior and middle cerebral arteries, respectively. The selection criteria for these regions were previously described in our earlier work [55]. To quantify the CoBF changes, the area above the CoBF curves (AOC, s*%) were also determined in the acute and subacute phases after CAO for each animal in both cerebrocortical regions.

### 4.4. Morphological Analysis of the Cerebrocortical Vasculature

Visualization of the cerebrocortical vasculature was achieved through transcardial perfusion of saline solution and black inks while under 2% isoflurane anesthesia. To begin, 10 mL of heparinized saline solution (10 IU/mL) was injected into the left cardiac ventricle. Subsequently, a 2 mL mixture of drawing ink (Koh-i-Noor Hardtmuth, České Budějovice, Czech Republic), endorsing ink (Interaction-Connect, Gent, Belgium), and distilled water in a 6:1:6 ratio was administered to the animals. Following this, the mice were decapitated, and their brains were placed in tubes containing a 4% formaldehyde solution for fixation. After a minimum of 24 h, pictures of the dorsal surface of the brains were captured using a digital camera and a Leica microscope (Leica MC 190 HD and Leica M80, respectively, both from Leica Microsystems, Wetzlar, Germany). The morphological analysis focused on the collaterals connecting branches of the anterior cerebral artery (ACA) and the middle cerebral artery (MCA) (Figure 1A). ImageJ software (Image J 1.5, NIH, Bethesda, MD, USA) was employed to analyze the acquired pictures. A blinded investigator utilized a micrometer etalon for calibration and performed calculations on the number and tortuosity index of the collaterals in both hemispheres. The tortuosity index was determined by dividing the vessel curve length by the line distance between the two ends of the vessel [68].

### 4.5. Statistical Analyses

To assess the normal distribution of the data, the Shapiro–Wilk test was employed. The results are presented as the arithmetic mean, accompanied by the standard error of the mean (SEM) or standard deviation (SD) when data are normally distributed and as median accompanied by interquartile range when data are not normally distributed. The significance levels were determined using Student’s unpaired t-test; whereas for experiments involving multiple variables, one-way or two-way repeated measures Anova, or Kruskal–Wallis tests were utilized, followed by Bonferroni’s or Tukey’s post hoc test, depending on the specific number of variables being analyzed and on the normality distribution of the data. Graphs and statistical analyses were performed using GraphPad Prism software (v.6.07; GraphPad Software Inc., La Jolla, CA, USA). A *p*-value of less than 0.05 was considered indicative of a statistically significant difference and denoted by an asterisk (*). For higher levels of significance, ** *p* < 0.01, *** *p* < 0.001, and **** *p* < 0.0001 were used to denote the corresponding *p* values.

## Figures and Tables

**Table 1:** Body parameters in NOS1 KO and NOS1/3 DKO animals compared to wild type controls.

	Wild Type	NOS1 KO	NOS1/3 DKO
body weight (g)	31.04 ± 0.57	26.13 ± 1.17 ****	26.04 ± 0.20 ****
heart weight (g)	0.153 ± 0.004	0.133 ± 0.008 *	0.121 ± 0.004 ****
heart weight/body weight (‰)	5.05 ± 0.14	5.29 ± 0.25	4.66 ± 0.18
tibial length (mm)	18.0 (17.9–18.0)	18.0 (17.0–18.1)	17.5 (17.5–18.0)
body weight/tibial length (g/mm)	1.698 ± 0.026	1.416 ± 0.058 ****	1.483 ± 0.024 ****
heart weight/tibial length (mg/mm)	8.549 ± 0.205	7.451 ± 0.362 *	6.892 ± 0.255 ***
brain weight (g)	0.456 ± 0.004	0.435 ± 0.007 *	0.430 ± 0.003 ***
brain weight/body weight (%)	1.503 ± 0.024	1.747 ± 0.082 **	1.653 ± 0.022 *
brain weight/tibial length (mg/mm)	25.48 ± 0.27	24.54 ± 0.59	24.49 ± 0.38

Mice lacking neuronal NOS (NOS1 KO) or both neuronal and endothelial NOS (NOS1/3 DKO) exhibited significantly lower body weight, heart weight, body weight/tibial length ratio, heart weight/tibial length ratio, and brain weight, whereas there was an increased brain weight/body weight ratio compared to wild type controls. The data are presented as either mean ± SE or median and interquartile range, depending on the normality of the data distribution. Statistical significance was calculated with one-way Anova test (* *p* < 0.05, ** *p* < 0.01, *** *p* < 0.001, **** *p* < 0.0001). The age of animals ranged between 12 and 17 weeks. The animal numbers were *n* = 10, 6, and 11 for the wild type, NOS1 KO, and NOS1/3 DKO groups, respectively.

**Table 2:** Arterial acid-base, blood gas, hematocrit, and electrolyte parameters.

	Control	NOS1KO	NOS1/3 DKO	L-NAME
pH	7.28 ± 0.04	7.26 ± 0.10	7.29 ± 0.06	7.24 ± 0.08
SBE (mmol/L)	−8.97 ± 0.88	−10.03 ± 2.00	−10.43 ± 1.89	−10.17 ± 0.94
HCO^3−^ (mmol/L)	17.96 ± 0.52	15.93 ± 1.46	18.40 ± 0.42	17.33 ± 0.83
pCO_2_ (mmHg)	39.8 ± 1.7	38.2 ± 2.7	40.3 ± 1.8	40.5 ± 1.9
pO_2_ (mmHg)	107.7 ± 2.29	119.7 ± 7.0	102.6 ± 2.0	112.2 ± 3.3
satO_2_ (%)	97.21 ± 0.27	97.80 ± 0.52	96.82 ± 0.41	96.95 ± 0.52
Hct (%)	41.4 ± 0.4	39.0 ± 0.8	41.5 ± 0.4	43.4 ± 0.9
Na^+^ (mmol/L)	155.6 ± 1.2	153.0 ± 1.2	156.5 ± 1.3	154.4 ± 0.9
K^+^ (mmol/L)	4.62 (3.88–4.98)	4.82 (4.70–4.93)	4.87 (4.37–4.48)	5.07 (4.30–6.20)
Ca^2+^ (mmol/L)	1.30 ± 0.04	1.30 ± 0.05	1.31 ± 0.03	1.24 ± 0.02

Arterial blood sampling from the femoral artery was conducted at the end of the experiment. The data are presented as either mean ± SEM or median and interquartile range, depending on the normality of the data distribution, as determined by the Shapiro–Wilk test. The sample sizes were *n* = 10, 6, 11, and 15 for the Control, NOS1 KO, NOS1/3 DKO, and L-NAME groups, respectively. No significant differences were observed among the experimental groups in these parameters.

## Data Availability

The datasets generated and analyzed during the current study are not publicly available due to the extensive mass of data, but the data are available from the corresponding author upon reasonable request.
